# Chemical Components and Pharmacological Activities of Terpene Natural Products from the Genus *Paeonia*

**DOI:** 10.3390/molecules21101362

**Published:** 2016-10-13

**Authors:** Dan-Dan Zhao, Li-Li Jiang, Hong-Yi Li, Peng-Fei Yan, Yan-Long Zhang

**Affiliations:** 1 Key Laboratory of Functional Inorganic Material Chemistry, Ministry of Education, School of Chemistry and Materials Science, Heilongjiang University, 74 Xue-Fu Road, Nan-Gang District, Harbin 150080, China; zhaodandan@hlju.edu.cn; 2 Key Laboratory of Molecular Biology of Heilongjiang Province, College of Life Sciences, Heilongjiang University, Harbin 150080, China; lljianghd@163.com; 3 Heilongjiang University Hospital, Harbin 150080, China; hylihd@163.com

**Keywords:** Genus *Paeonia*, terpene, chemical components, pharmacological activities

## Abstract

*Paeonia* is the single genus of ca. 33 known species in the family *Paeoniaceae*, found in Asia, Europe and Western North America. Up to now, more than 180 compounds have been isolated from nine species of the genus *Paeonia*, including terpenes, phenols, flavonoids, essential oil and tannins. Terpenes, the most abundant naturally occurring compounds, which accounted for about 57% and occurred in almost every species, are responsible for the observed in vivo and in vitro biological activities. This paper aims to give a comprehensive overview of the recent phytochemical and pharmacological knowledge of the terpenes from *Paeonia* plants, and enlighten further drug discovery research.

## 1. Introduction

Natural products contribute significantly to drug discovery research with a rich source of compounds and provide inherently large-scale of structural diversity than synthetic compounds [1]. The genus *Paeonia* belongs to the family *Paeoniaceae* and consists of about thirty-three known species [2]. The roots of *P. suffruticosa*, *P. obovata*, and *P. lactiflora* are important sources of crude drugs in traditional Chinese medication with activities of nitric oxide production inhibitory effects [3,4], anti-tumor activity [5,6], anti-inflammatory effects [7], anti-influenza virus [8], hematopoietic effects [9], anti-aggregatoryand and anti-coagulative effects [10].

## 2. Plant Distribution

The genus *Paeonia* naturally distributes in the cold and temperate areas of the Northern Hemisphere. They are mainly distributed in Asia and Europe, and only a few native to Western North America. A total of 11 species are found in China, with wide distribution in southwestern and northwestern areas, central China, northern and northeastern China [11].

In detail, *P. emodi* grows in the western Himalayas between Nepal and Pakistan [12]. *P. obovata* naturally distributes in forests ranging from deciduous broad-leaved to coniferous forests and may be found at an altitude from 200 m to 2800 m. In China, it occurs in Anhui, Gansu, Guizhou, Hebei, and Heilongjiang et al. It also grows in Korea, Russia and Japan [13]. In addition, *P. lactiflora* occurs in northern and northeastern China, Korea, Japan, Mongolia, Russia Far East and Siberia [14]. *P. veitchii* distributes in western China including Shanxi, Gansu, Ningxia, Qinghai, Sichuan and the eastern rim of Tibet [15]. *P. suffruticosa* grows in Central to Northern China including Tongling, Heze, Luoyang, Pengzhou, and Beijing [16]. *P. delavayi* is endemic to southwestern China, where its habitat is limited to Sichuan, Yunnan and the very South-East of Tibet [17].

## 3. Chemical Constituents

**Figure 1:**
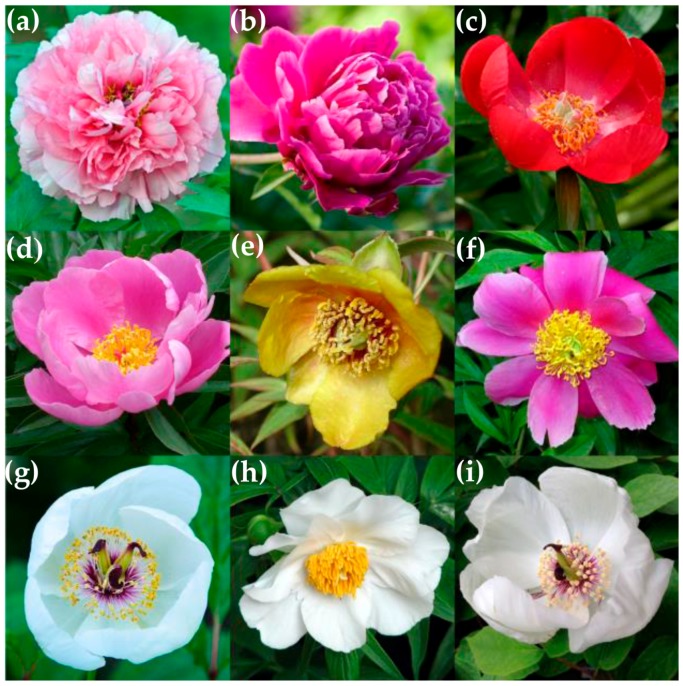
The flowers of the chemically investigated nine species of the genus *Paeonia*. (**a**) *P. albiflora*; (**b**) *P. delavayi*; (**c**) *P. emodi*; (**d**) *P. japonica*; (**e**) *P. lactiflora*; (**f**) *P. obovata*; (**g**) *P. peregrina*; (**h**) *P. suffruticosa*; (**i**) *P.* *veitchii*. (https://it.wikipedia.org/wiki/Paeonia).

**Figure 2:**
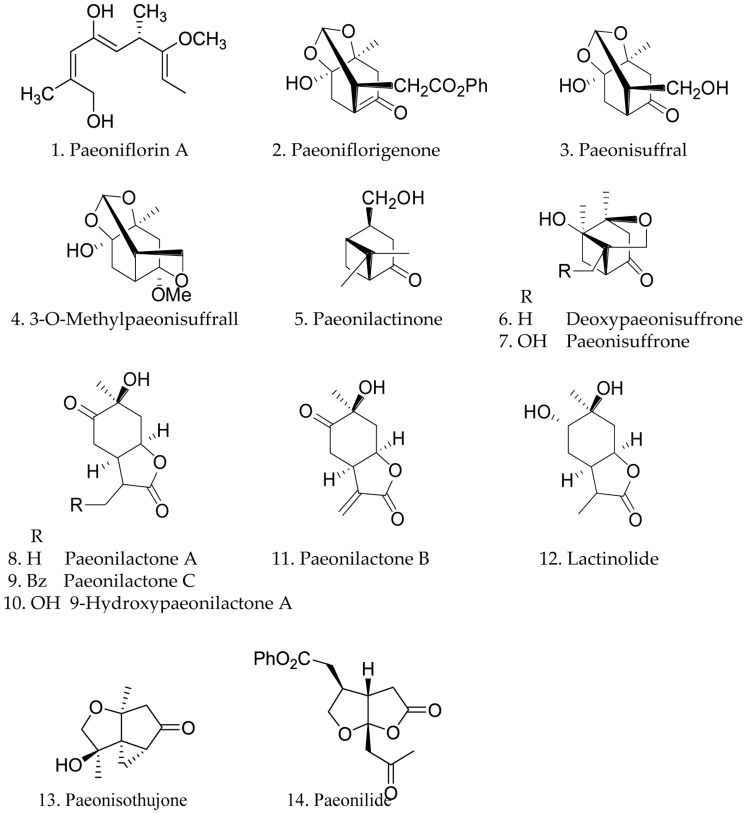
Chemical structures of monoterpene.

**Figure 3:**
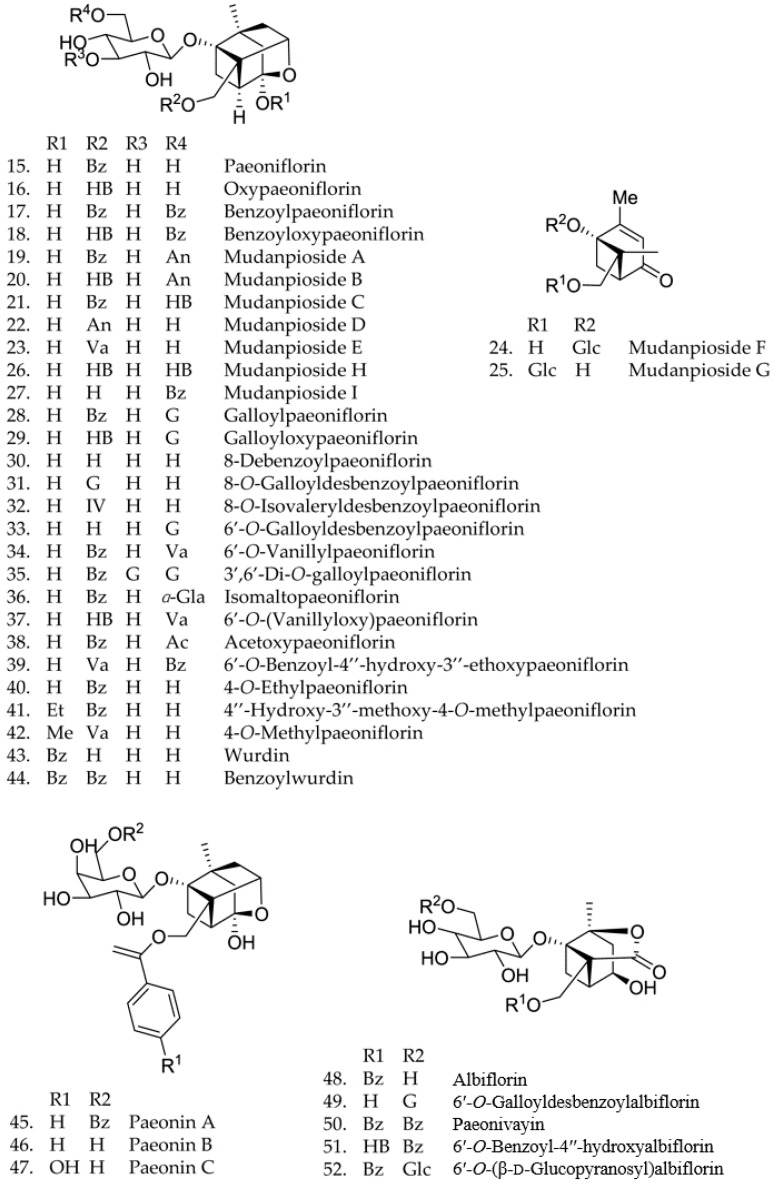

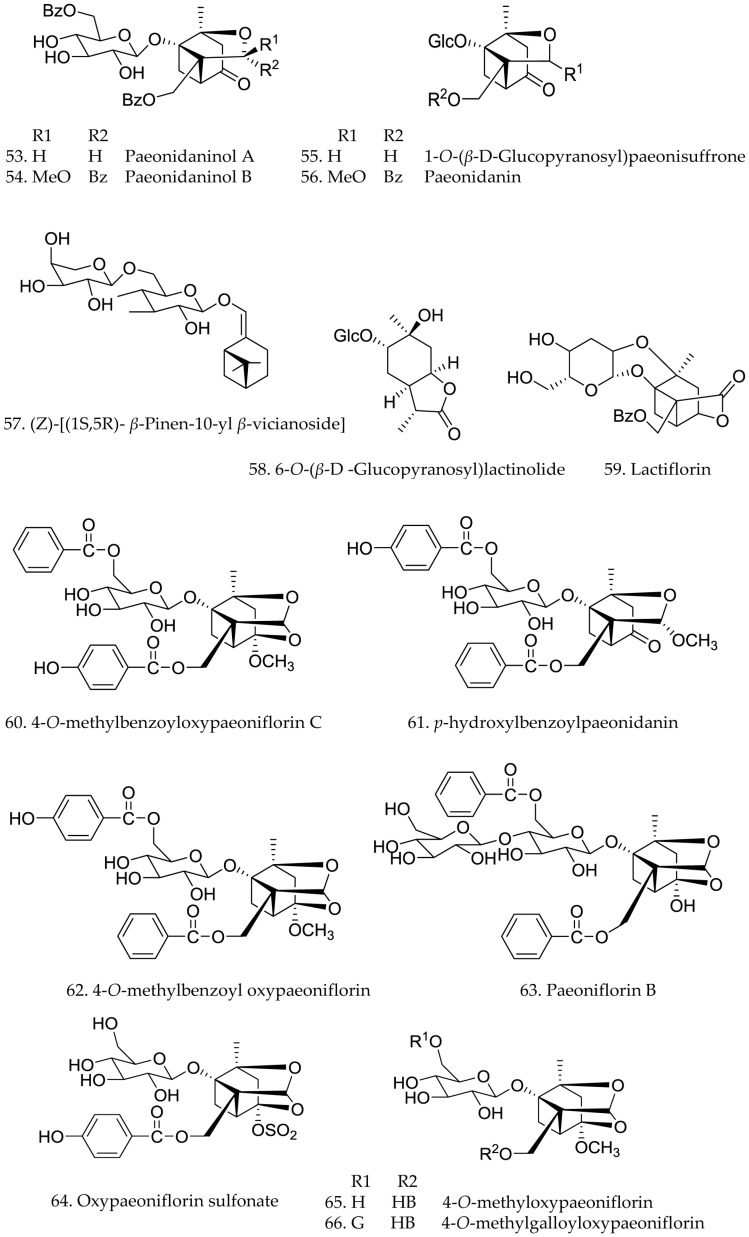

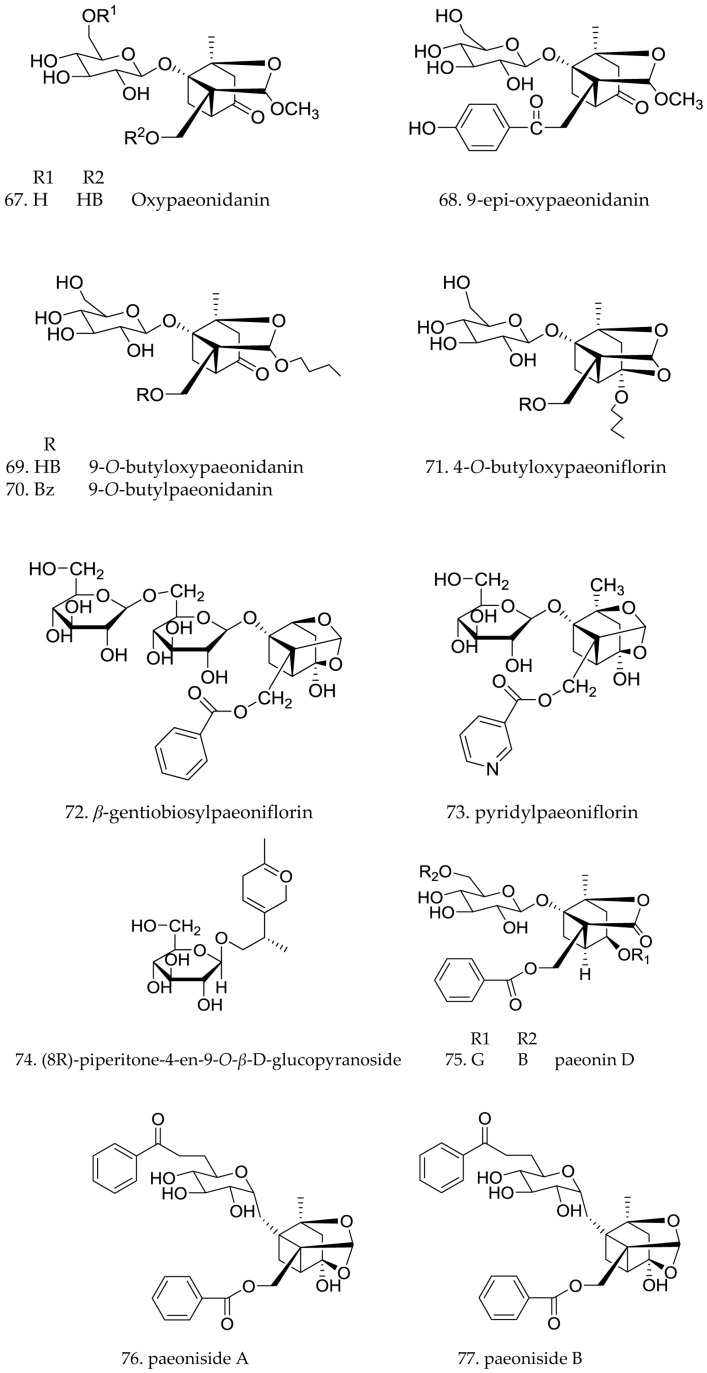
Chemical structures of monoterpene glycosides.

**Figure 4:**
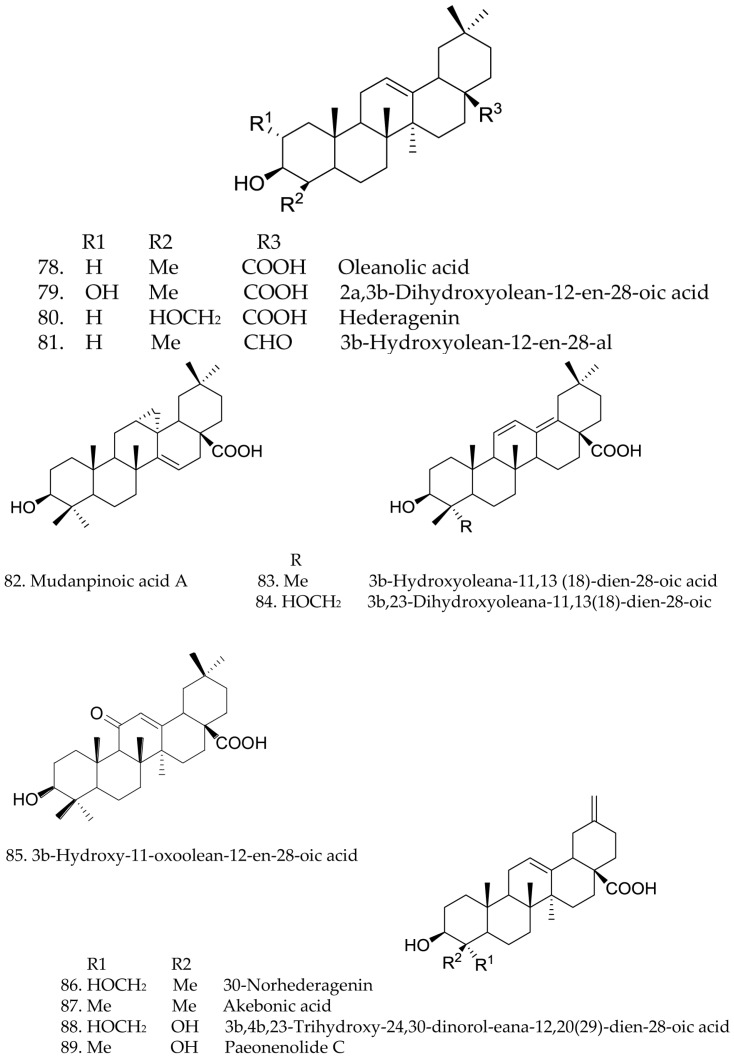

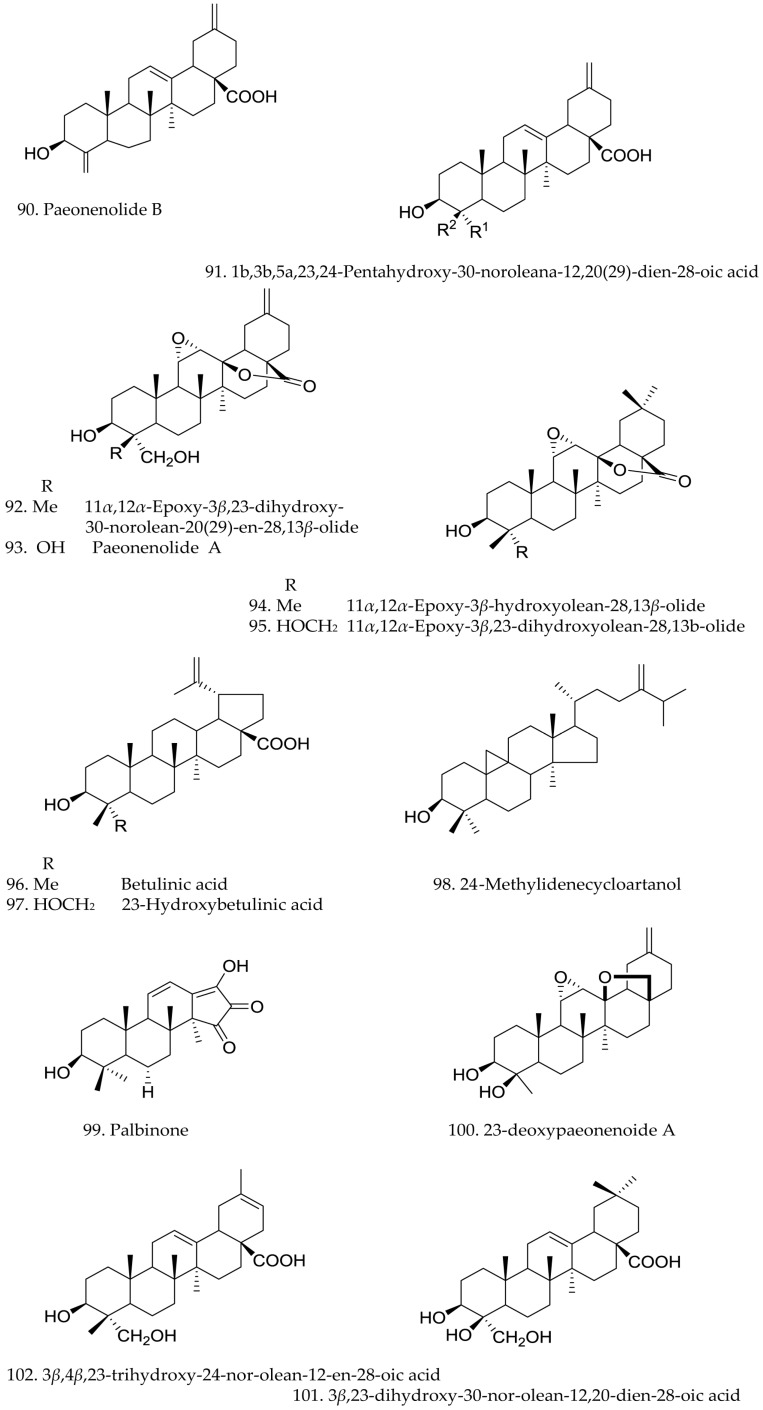

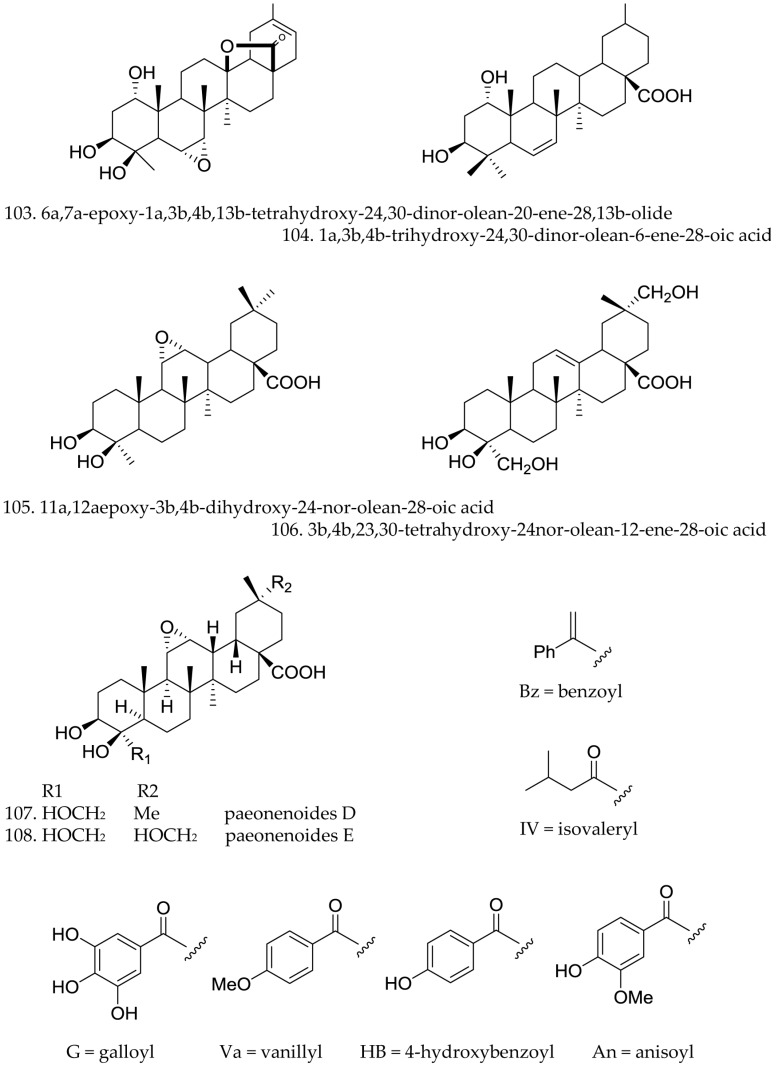
Chemical structures of triterpenes.

The present chemical studies of *Paeonia* plants were more focused on the composition of the roots and less on other parts. Since 1753 [18], more than 180 compounds have been isolated. Nine species of the genus *Paeonia* have been chemically investigated, including *P. albiflora*, *P. delavayi*, *P. emodi*, *P. japonica*, *P. lactiflora*, *P. obovata*, *P. peregrina*, *P. suffruticosa*, *P. Veitchii*. Their flowers are shown below in Figure 1a–i. The skeletons of terpenoid compounds from this genus included monoterpenes, monoterpene glycosides and triterpenes. Their structures and names are summarized below (structures 1–108 and Figure 2, Figure 3 and Figure 4). As shown, monoterpene glycosides are the important components in the genus *Paeonia*.

### 3.1. Monoterpenes

Fourteen monoterpenes, 1–14, were isolated from *Paeonia* species. Paeoniflorin A (1) was obtained from the root cortex *P. suffruticosa* [19]. Paeoniflorigenone (2), one of the main bioactive constituents, was found in three species, *P. suffruticosa*, *P.* *peregrina*, and *P. albiflora* [20]. Most monoterpenes were obtained from *P.* *suffruticosa* [21]. Three *p*-menthane monoterpenes, paeonilactone A–C (8, 9, and 11, resp.), were obtained from *P. albiflora*. In 1996, Paeonilactinone (5) and Lactinolide (12) were reported from *P.* *lactiflora* [22]. Later, paeonilide (14) were found in *P. delavayi* [23]. Their structures, 1–14, are shown below, and their names are collected in Figure 2.

### 3.2. Monoterpene Glycosides

A total of 58 monoterpene glycosides, 15–77, have been isolated from *Paeonia* species. Compounds 15–57 are pinane type derivatives with a variety of substituents [8,22,24,25,26,27,28,29,30,31,32,33,34,35,36,37,38,39,40,41,42,43]. Later, 6-*O*-(β-d-glucopyranosyl)lactinolide (58), and lactiflorin (59) were obtained from *P. lactiflora* [44]. *Paeonia* species are a rich source of monoterpene constituents possessing a “cage-like” pinane skeleton, which are found as the main biologically active compounds.

In 2012, 11 monoterpene glycosides, including, 4-*O*-methy-lmoudanpioside C (60), *p*-hydroxylbenzoyl-paeonidanin (61), 4-*O*-methylbenzoyl oxypaeoniflorin (62), paeoniflorin B (63), oxypaeoniflorin sulfonate (64), 4-*O*-methyloxypaeoniflorin (65), 4-*O*-methylgalloyloxy paeoniflorin (66), oxypaeonidanin (67), 9-epi-oxypaeonidanin (68), 9-*O*-butyloxypaeonidanin (69), 9-*O*-butylpaeonidanin (70), and 4-*O*-butyloxypaeoniflorin (71), were obtained from the ethanol extract of *P. suffruticosa Andrews* [4]. They equally have “cage-like” pinane skeleton. In addition, in 2012, β-gentiobiosylpaeoniflorin (72), pyridylpaeoniflorin (73), (8*R*)-piperitone-4-en-9-*O*-β-d-gluco-pyranoside (74) are isolated from *P. suffruticosa* [45]. In 2014, a new monoterpene glucoside, paeonin D (75), were obtained from *P. lactiflora* [46]. In the same year, paeoniside A (76) and paeoniside B (77) were isolated from *P. suffruticosa* [47]. Their structures, 15–77 are shown below, and their names are collected in Figure 3.

### 3.3. Triterpenes

A total of 30 triterpenes, 78–108, have been reported from various *Paeonia* species [44,48,49,50,51,52,53]. Among them, compounds 78–80, 83–86, and 94–97 were isolated from the callus tissues of *P. suffruticosa*, *P. lactiflora*, and *P. japonica* [47,48,54]. Later, four novel 24,30-dinortriterpenoids, 88–90 and 93, were only found in *P. delavayi* [17]. In 2011, three noroleanane triterpenes 100–102 were obtained from *P. rockii* [50]. In 2012, four noroleanane triterpenes 103–106 were obtained from *P. emodi* [55]. In addition, in 2016, two new nortriterpenoids, paeonenoides D (107) and paeonenoides E (108), were obtained from *P. lactiflora* [56]. Their structures, 78–108 are shown below, and their names are collected in Figure 4.

## 4. Biological Activities

### 4.1. Inhibitors of Nitric Oxide Production

Three compounds (1, 17 and 62) was showed significantly suppressed nitric oxide production [4]. Paeoniflorin (15) inhibiting inflammation and inducible nitric oxide synthase signaling pathways, and ameliorates acute myocardial infarction of rat [3]. Compounds 107, 108 were showed inhibitory effects against nitric oxide production in LPS-induced RAW246.7 macrophages [56].

### 4.2. Anti-Tumor Activity

A few of the compounds showed significant cytotoxicity against a panel of human cancer cell lines. Compound 95 against MCF-7, HT-29, M-14 [57]; Compounds 104–106 against A549, HCT116, HL-60, ZR-75-30; Compounds 104–106 against HL-60, HCT116 and ZR-75-30 [58]; Compounds 107, 108 against Hep-G2, SK-OV-3, HL-60 [56].

### 4.3. Anti-Inflammatory Effects

Palbinone (99) have a strong inhibitory anti-inflammatory effect NF-κB signal pathway [52]. Paeoniflorin (15) may ameliorate acute renal injury following ANP in rats by inhibiting inflammatory responses and renal cell apoptosis, due to the p38-MAPK and NF-κB [59]. In addition, in 2016, Zhihong M. et al. reported it to have liver protective and anti-inflammatory effects in HCF diet-induced NASH rats, associated with inhibition of the ROCK and NF-κB in the NASH liver [7]. In addition, paeoniside A (76) inhibited against cyclooxygenase-1 (COX-1) and cyclooxygenase-2 (COX-2) enzymes [47].

### 4.4. Anti-Oxidative Effects

Compounds 28 and 29 have potent radical-scavenging remarkable effects on DPPH, and compounds 16 have a weak radical-scavenging effect [60]. It is also demonstrated paeonins A (45) and paeonins B (46) exhibited inhibitory activities against lipoxygenase [40]. Paeonin C (47) has potent inhibitory potential against lipoxygenase in a concentration-dependent fashion [61]. Compounds 33 demonstrated a significant scavenging capacity against the DPPH free radical, ROS, the superoxide anion radical, and the hydroxyl radical [62].

### 4.5. Anti-Aggregatory and Anti-Coagulative Effects

Paeonilide (14) selectively inhibited the platelet aggregation induced by platelet activating factor [23]. Koo Y., et al. reported that paeoniflorin (15) and benzoylpaeoniflorin (17) have obvious inhibitory effect on collagen, endotoxin and adenosine diphosphate (ADP)-induced blood platelet coagulation, but not on blood aggregation in vitro. In addition, compounds 15 and 19 exhibited blood coagulation-inhibitory activity in vivo [10].

### 4.6. Sedative and Analgesic

Paeoniflorin (15) could modulate sleep behaviors and the mechanisms involved, and increased NREM sleep by inhibiting the histaminergic system via A1 receptors [63]. In addition, it could inhibit formalin-induced nociceptive behavior in mice, these effects may be might be associated with modulation of NMDA receptors, specifically the NR2B subunit [64]. Shimizu et al. found that paeoniflorigenone (2) produced a blocking effect on neuromuscular junction in phrenic nerve diaphragm preparations of mice [65]. In addition, paeonilactone C (9) was showed to suppress stimulated muscle twitchings of frog sciatic nerve-sartorius muscle [66].

### 4.7. Other Activities

Paeoniflorin (15) and 8-debenzoylpaeoniflorin (30) showed a blood sugar lowering effect in streptozotocin-treated rats [67]. 1-*O*-(β-d-Glucopyranosyl)paeonisuffrone (55) was found to inhibit histamine release from rat peritoneal exudate cell-induced antigen-antibody reaction [22]. Compound 52 was found to have a direct stimulatory effect on bone formation in vitro and may contribute to the prevention for osteoporosis [42]. Paeoniflorin (15) has previously been reported to alleviate hepatic fibrosis. Paeoniflorin was found to effectively prevent renal interstitial fibrosis [68]. Paeoniflorin (15) has sedative, hypotensive, and weak anti-inflammatory effects, and a preventive effect on stress ulcer [69]. Compound 89 showed inhibitory activity against β-glucuronidase [70].

## 5. Conclusions

*Paeonia* is the only genus in the family *Paeoniaceae* and has significant medicinal importance in traditional Chinese medicine. Researchers have different views on the number of species that can be distinguished ranging from 25 to 40 [71,72], although the current consensus is thirty-three known species. Based on data available, this paper summarizes three types of terpene compositions and exhibited their bioactivities such as inhibitors of nitric oxide production, anti-tumor activity, anti-inflammatory effects, anti-oxidative effects, anti-aggregatoryand and anti-coagulative effects, sedative and analgesic activity. Taken together, the compounds from *Paeonia* plants have a great potential to be used as new chemical drugs in future. However, only nine species of the genus *Paeonia* have been chemically studied, it should be urgent to study other species for more potential bioactive components. In addition, the relationships between the species also need to be further clarified.
